# The prevalence, temporal and spatial trends in bulk tank equivalent milk fat depression in Irish milk recorded herds

**DOI:** 10.1186/s13620-017-0092-y

**Published:** 2017-05-18

**Authors:** Catherine I. Carty, Alan G. Fahey, Morgan R. Sheehy, Steve Taylor, Ian J. Lean, Conor G. McAloon, Luke O’Grady, Finbar J. Mulligan

**Affiliations:** 1 School of Veterinary Medicine, University College Dublin, Dublin, Ireland; 2 School of Agricultural Food Science and Nutrition, University College Dublin, Dublin, Ireland; 3 Devenish Nutrition, Lagan House, 19 Clarendon Road, Belfast, Northern Ireland; 4 Scibus Consultancy, 2 Broughton Street, Camden, NSW Australia

**Keywords:** Milk fat, Milk fat depression, Milk solids, Dairy cows

## Abstract

**Background:**

Milk fat is important in terms of economic value and in its potential to provide information concerning cow diet and health. Under current milk payment schemes in Ireland farmer income is directly linked to milk fat production.

**Methods:**

A descriptive analysis of milk fat depression (MFD) as calculated from test day milk recording data across all milk recording herds from 2004 to 2014 was undertaken. A dataset of 17 million test day records was used to calculate the prevalence of MFD in Irish milk recorded herds and to create a graphical description of the major descriptive epidemiological trends in milk fat depression in time and space in Ireland. The bulk tank equivalent (BTE) for test day milk fat was calculated for each herd and for cohorts of cows within herds using the formula; BTE milk fat = sum test day fat kg/sum test day milk kg. Milk fat depression was defined as BTE <3.3% milk fat and BTE > 3.2% milk protein.

**Results:**

The annual prevalence of MFD decreased significantly over time in a linear manner until 2014. Across all years the highest prevalence of MFD occurred in April or May. The highest prevalence occurred most commonly in May, with 9.1% of herds experiencing MFD in 2014. The highest prevalence of MFD in autumn calved cohorts occurred at 181–210 days in milk whereas it occurred at days 61–90 in milk in spring calving cohorts. The stage of lactation for the most common occurrence of MFD in both the spring and autumn cohorts corresponded with the month of May. There were some notable spatial patterns regarding variations in prevalence of MFD across the country. Cohorts of cows with the highest genetic values for milk yield had the highest prevalence of MFD whereas cohorts of cows with the highest breeding values for milk fat percent had the lowest prevalence of MFD.

**Conclusions:**

A subpopulation of Irish herds experienced the condition of MFD. Descriptive analysis suggested spatial, temporal and animal level associations. This condition warrants further investigation.

**Electronic supplementary material:**

The online version of this article (doi:10.1186/s13620-017-0092-y) contains supplementary material, which is available to authorized users.

## Background

Milk fat is an important component of milk from a nutritional perspective. For dairy processors, who buy the milk from the farmer and set milk price, higher levels of milk fat are desirable for its economic value, particularly for value added dairy products. For dairy farmers milk payment schemes have evolved in Ireland so that payment is for kilograms of fat and protein produced, with a volume charge due to processing costs, subtracted for each litre of milk supplied [1, 2]. Although milk protein is more valuable than milk fat, both are important components of farm income. The Irish dairy industry produces commodity products such as butter, milk powder and cheese, thus milk fat and protein are essential in the manufacturing of these products. There are differences in how efficiently milk solids are produced between farms. Cows that can most efficiently convert grass to milk solids are the most profitable in pasture based systems, such as the farming system most commonly encountered in Ireland [3].

The normal milk fat percentage for Holstein herds has been defined as between 3.4 and 4.0% with some seasonal variation [4]. Milk fat depression (MFD) or low milk fat syndrome is defined as reduced milk fat yield in the presence of normal or expected milk yield or yield of other milk components such as milk protein [5, 6]. In some cases this depression may be up to a 50% reduction in milk fat yield with a greater decline in the *de novo* synthesised portion of milk fatty acids [6]. Other definitions used include milk fat percentage below 3.2% for Holstein herds [4], or a decrease in herd milk fat percentage of more than 0.4% for a period of ten days or more [5]. In addition to these herd level definitions within herd targets have been proposed. It has been suggested that no more than 10% of any group of cows, especially after 70 days in milk, should have a milk fat test of below 2.5% [4].

The physiology of milk fat production in cattle has been widely investigated in many studies. Milk fat is produced partially *de novo* in the mammary gland by epithelial cells from precursors such as acetate and beta-hydroxybutyrate which are absorbed from the blood. The remainder of milk fat is produced from mammary uptake of preformed fatty acids originating from the gastro-intestinal tract [7, 8]. The diet of the cow has a profound effect on milk fat as the concentration and yield of milk fat are driven primarily by the nutrition of the cow [9]. However the percentage fat in milk can also vary depending on breed/genetics, stage of lactation, season, and the health of the cow [10]. Breed has an important influence on milk fat with Jersey and Guernsey breeds having higher milk fat than Holsteins. Genetic breeding values of milk fat are present in most selection indices. Stage of lactation also affects milk fat; as mobilisation of body fat reserves can occur if cows are in negative energy balance in early lactation, resulting in higher milk fats, mobilisation also reflects how the health of the cow can influence milk fat [7]. Milk fat percentage will normally increase toward the end of lactation when milk yield is lower [10]. Seasonal effects of milk fat production follow that milk fat percentage tends to be higher in winter and lower in summer with different patterns in the fatty acid composition depending on season [11–13]. Furthermore, timing of sample collection during milking and the inter-milking interval can influence milk fat percentage [5]. Milk fat percentage can vary with differing testing equipment and methods or due to sampling error. Known patterns of variability include time of day; milk fat is expected to be higher in the morning [14] and timing of sample collection during milking, with higher fat concentrations present towards the end of milking [10].

A biohydrogenation theory is accepted as the most plausible physiological basis for MFD [9]. Partial ruminal biohydrogenation of dietary polyunsaturated fatty acids (PUFA), leads to the production of isomers of conjugated linoleic acid (CLA). Particular isomers of CLA such as trans-10, cis-12 CLA are absorbed and act at the level of the mammary gland to signal a decreased expression of mammary lipogenic enzymes [15]. A reduction in ruminal pH may result in altered microbial processes involved in the biohydrogenation of PUFA’s and the production of the causative CLA isomer [9]. However, the rumen conditions required for MFD are not fully understood [9]. A continuous substrate supply of dietary PUFA allows the MFD cycle to proceed [9]. As the predominant milk production system in Ireland is based on grazed grass, high concentrations of polyunsaturated fatty acids in grass, may precipitate MFD [15, 16].

Exploring the epidemiological presentation of MFD will allow, for the first time, to document the severity of the problem in Ireland and may yield insights into important risk factors to focus further research. There is little information on the prevalence of MFD in grazing herds in Northern Europe. Therefore, the objective of this study was to estimate the prevalence of, and profile the spatial and temporal patterns of MFD in Irish Holstein/Friesian milk recorded herds.

## Methods

Holstein and Friesian test day milk records between the years 2004–2014, were obtained for all individual cows from the Irish Cattle Breeding Federation (ICBF) database. Test day records included milk yield, fat and protein yield and percentages, cow breed, breeding values as measured by economic breeding index (EBI) and predicted transmitting ability (PTA) values for relevant traits (milk yield, milk fat and protein percentages). Herd location at a county level was also available for each unique herd. The EBI used in Ireland is a single figure profit index used for breeding. The EBI is a weighted figure made of seven sub-indices including production and fertility. The PTA is the heritable phenotypic difference in certain genetic traits relative to a base cow [17, 18].

Data analysis and visual presentation of results were performed using R Core Team [19]. The test day records dataset contained test day records on 1.05 million unique cows. It is estimated that approximately one third of Irish dairy farmers milk record [20]. Animals where the main breed was Holstein or Friesian were retained. The final dataset had 9,337 unique herds, with a mean six milk recordings per year across all 11 years.

Data were analysed for outliers and the following data edits were conducted; records that were missing test day (TD) milk yield, TD milk fat kgs, TD milk protein kgs, EBI, herd identification or test date were removed from the dataset. Test day records that were ± 3 standard deviations from the mean of TD milk yield, TD milk fat or protein yield were omitted. In addition, TD milk yields <4 kg, as well as TD records for animals of parities >10 were removed. Only records of cows recorded once per month and at least four times per year were retained. Days in milk (DIM) was calculated for all cows by subtracting the recorded calving date from the sampling date, and all records <1 and > 305 days in milk were omitted. The final working dataset contained 17,312,806 test day records.

As the focus of this study was the composition of milk supplied from farms it was therefore, necessary to calculate a yield weighted average or bulk tank equivalent (BTE) to represent the milk fat composition of the bulk tank on the day of the milk recording. Herd level variables were created for herds in the dataset based on individual test records for all months in which they were milk recorded. The test variables BTE milk fat and milk protein percentages were created for each herd on each test day by dividing the sum of the milk fat kg and the sum of the milk protein kg by the sum of the milk kg for that herd and converting to a percentage.

$$ \mathrm{B}\mathrm{T}\mathrm{E}\ \mathrm{Milk}\ {\mathrm{Fat}}_j=\frac{{\displaystyle {\sum}_{i=1}^n}\mathrm{test}\ \mathrm{day}\ \mathrm{milk}\ \mathrm{fat}\ \mathrm{kg}}{{\displaystyle {\sum}_{i=1}^n}\ \mathrm{test}\ \mathrm{day}\ \mathrm{milk}\ \mathrm{kg}} $$

Where n is the number of i-cows in the j-th cohort or herd.

A “herd” was defined as all cows with the same unique herd identifier on each monthly test day across the dataset. For the purpose of this study, a definition of MFD encompassing more than one criterion was used; MFD was suggested to exist if BTE milk fat was below 3.3% without a simultaneous inappropriate reduction in milk protein percentage. MFD in herds or cohorts was defined as a BTE test day fat percentage below 3.3% and a BTE test day protein percentage of greater than 3.2%. Herds where BTE test day milk fat was < 3.3% and BTE milk protein was <3.2% were classified as low milk solids herds and were therefore not included in our definition of MFD. The binary outcome used was MFD = 1 if the definition criteria were met, that is BTE milk fat <3.3% and BTE milk protein >3.2% for both herd and cohort analysis.

Cohort level analysis was also conducted; these cohorts were created from subgroups of cows within herds based on parity (1,2,3,4,5+), calving season and DIM; DIM were divided into 30 day subgroupings. Calving season was defined as either autumn (cows calving in September to December inclusive), spring (cows calving in January to May inclusive) or other (cows calving in months June – August inclusive). Milk yield and fat percentage PTA’s were binned into quintiles. Cohorts of cows within herds were assigned to each of these quintiles for analysis. Spatial patterns were visualised for each county in Ireland by mapping the prevalence of MFD in Ireland over time and by calving season. Only cohort sizes of five or more animals were used in any of the cohort analyses. BTE milk fat and BTE milk protein was calculated for each cohort using the same method as for herd level analysis.

Prevalence of MFD at herd level was calculated for each month across each year of the data. This data was not aggregated and all herd test day records for each month were used to calculate herd level prevalence. Next, prevalence of MFD at cohort level was calculated and plotted for calving season by month, calving season by days in milk, parity by month, quintiles of PTA milk yield by month and quintiles of PTA milk fat percent by month. For this analysis, data were aggregated across all 11 years of the data. For each month, the prevalence of MFD was calculated by total number of recordings with MFD divided by the total number of recordings across all the years of the dataset. The 95% confidence intervals were also calculated for each prevalence and p values calculated for any direct comparisons.

To explore the statistical associations between year and month on herd level prevalence of MFD, two mixed effects logistic regression models were constructed. In model 1, a mixed effects logistic regression model was performed with herd included as a random effect and year and month as fixed effects. A second mixed effects logistic regression model (model 2) was constructed using year as a fixed effect and herd nested within month as a random effect. The regression coefficients from this model were used to predict the probability of MFD for each year accounting for herd as a random effect [21]. Probabilities were plotted to display the trend in herd level prevalence of MFD over time and a line of best fit was fitted using the most appropriate R squared value.

## Results

Over the 11 years of the dataset there was a reduction in the total number of herds milk recorded per year from 45,818 herd test days in 2004 to 32,393 herd test days in 2014 The mean number of times that herds were milk recorded per year decreased from 8 to 6, however the modal number recordings per year was four for the last nine out of 11 years. Some of the descriptive tables are available in Additional files 1, 2 and 3.

**Fig. 1:**
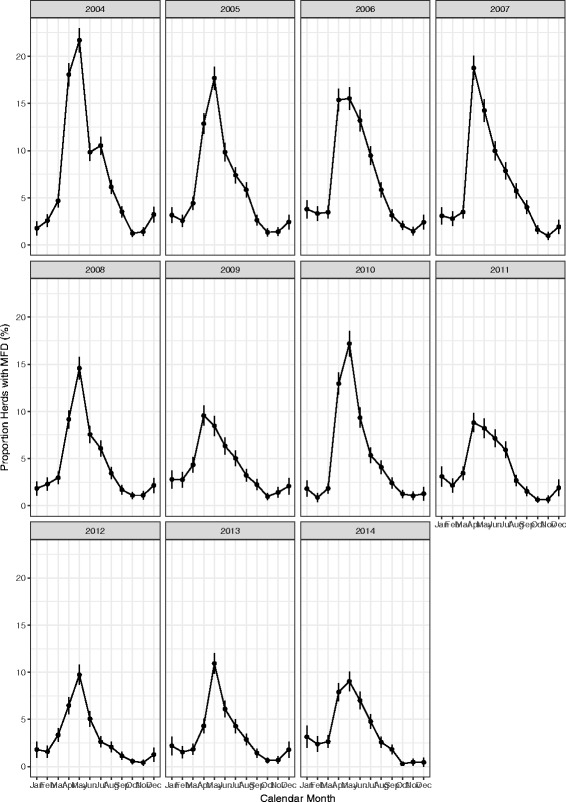
Prevalence of MFD of milk recorded herds with MFD by month over 11 years of milk recording data

Temporal patterns of MFD are shown in Fig. 1. The highest prevalence of MFD in 2014 was lower than the highest prevalence of MFD in 2004 (*p* < 0.001); 9.1% (95% confidence interval; 8.1, 10.1%) and 21.7% (95% confidence interval; 20.5, 22.9%) respectively. The duration of MFD also differed from year to year, with the prevalence of MFD returning to low levels (≤5%), by autumn in most years. The highest prevalence of MFD occurred most often in May, with the highest prevalence occurring in the month of April in 2007, 2009 and 2011. The lowest prevalence occurred in October or November across each of the 11 years.

**Fig. 2:**
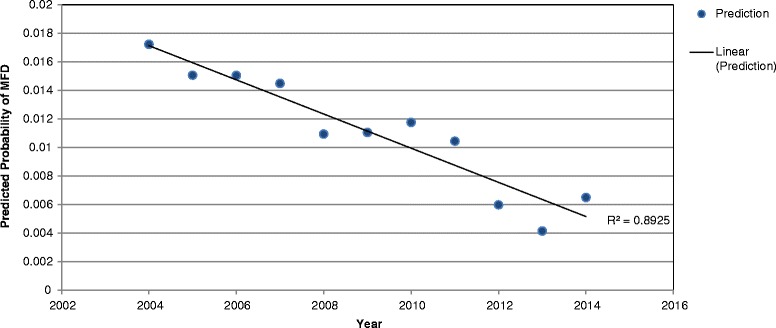
Trend in decreasing herd level prevalence of MFD from 2004 to 2014

The outputs from model one are shown in Table 1. The odds of herds having MFD in May were 27 times the odds of having MFD in November (*p* < 0.001). All years had a lower prevalence of MFD than 2004 with the odds of having MFD in 2014 0.37 times the odds of having MFD in 2004 (*p* < 0.000). The results of model two are shown in Table 2. The predicted probabilities of herd level prevalence of MFD across the 11 years of data are presented in Fig. 2. A line of best fit fitted using the best R squared value displayed a linearly decreasing prevalence of MFD between 2004 and 2014.

**Table 1:** Multivariable logistic regression estimates of odds ratios of different months having MFD, including herd as a random effect

Month	Odds Ratio	z	*p* value	95% Conf Interval	
Jan	2.24	0.31	0.00	1.70	2.93
Feb	3.04	0.4	0.00	2.36	3.93
Mar	5.18	0.62	0.00	4.09	6.56
Apr	23.43	2.66	0.00	18.75	29.27
May	27.01	3.06	0.00	21.63	33.73
Jun	12.39	1.43	0.00	9.88	15.53
Jul	8.38	0.98	0.00	6.66	10.53
Aug	5.55	0.66	0.00	4.39	7.00
Sep	2.75	0.35	0.00	2.14	3.53
Oct	1.27	0.18	0.09	0.96	1.67
Nov	Reference				
Dec	1.86	0.27	0.00	1.40	2.47
Year					
2004	Reference				
2005	0.73	0.38	0.00	0.66	0.81
2006	0.77	0.04	0.00	0.69	0.86
2007	0.79	0.04	0.00	0.71	0.88
2008	0.57	0.03	0.00	0.51	0.64
2009	0.54	0.03	0.00	0.48	0.61
2010	0.62	0.04	0.00	0.55	0.7
2011	0.54	0.03	0.00	0.47	0.61
2012	0.29	0.02	0.00	0.25	0.33
2013	0.2	0.02	0.00	0.17	0.24
2014	0.37	0.03	0.00	0.32	0.42

**Table 2:** Result of forward prediction from multivariate logistic regression model showing the mean probability of MFD each year accounting for random effect of herd

Year	Mean Predicted Probability of MFD	Standard Error	95% Conf. Interval	
2004	0.0172	0.000	0.017	0.018
2005	0.0151	0.000	0.015	0.016
2006	0.0150	0.000	0.014	0.016
2007	0.0145	0.000	0.014	0.015
2008	0.0109	0.000	0.010	0.011
2009	0.0110	0.000	0.011	0.011
2010	0.0118	0.000	0.011	0.012
2011	0.0104	0.000	0.010	0.011
2012	0.0060	0.000	0.006	0.006
2013	0.0041	0.000	0.004	0.004
2014	0.0065	0.000	0.006	0.007

**Fig. 3:**
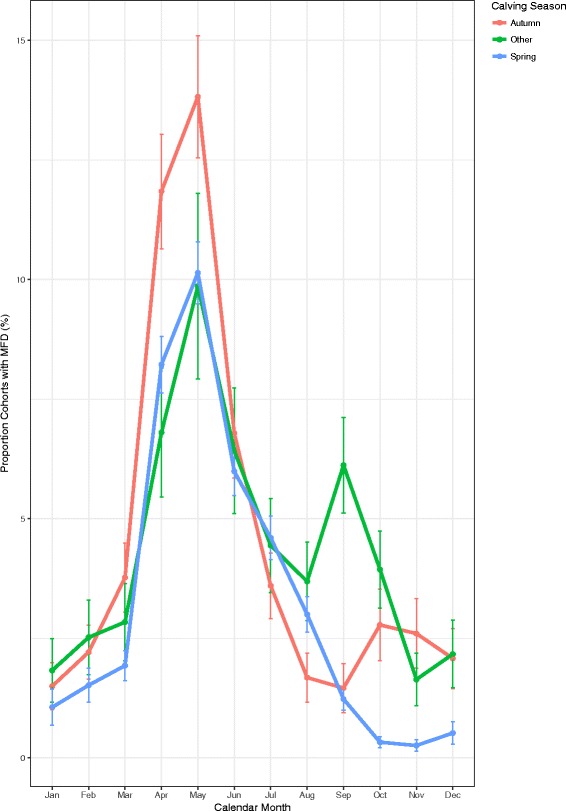
Monthly prevalence of MFD across within-herd cohorts of cows by calving season with error bars showing 95% confidence intervals

The proportion of cohorts defined by calving season and calendar month with MFD show a similar temporal trend over the course of the year (Fig. 3). Groups of autumn calved cows within herds had a higher proportion of MFD from March to June, across the 11 years. The highest prevalence of MFD in autumn calved cohorts was higher than the highest prevalence in the spring calved cohorts (*p* < 0.01). In addition there was a reduction in the peak prevalence between autumn calved cohorts and the cohorts defined as other (*p* = 0.02). The highest prevalence (95% confidence interval) of MFD was 13.8% (12.5, 15.1) for autumn calved cohorts of cows, compared to 10% (9.4, 10.6) in spring calved cohorts. The highest prevalence occurred in May for both groups.

**Fig. 4:**
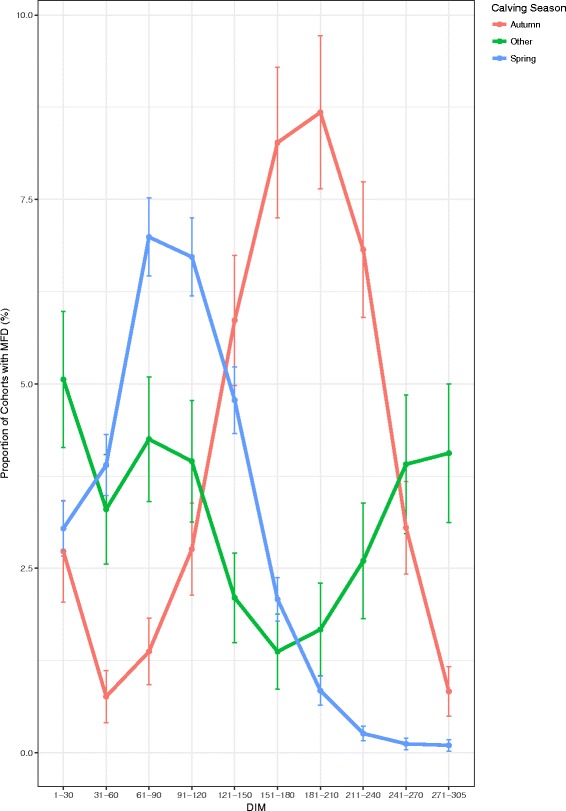
Monthly prevalence of MFD across within-herd cohorts of cows by calving season and stage of lactation; days in milk (DIM) with error bars showing 95% confidence intervals

Figure 4 shows that in autumn calving cohorts of cows, the greatest prevalence 8.7% (7.6, 9.7) of MFD occurred at 181–210 DIM. In contrast, in the spring calved cohort of cows, the highest prevalence, 7% (6.5, 7.5) occurred at days 61–90 DIM.

**Fig. 5:**
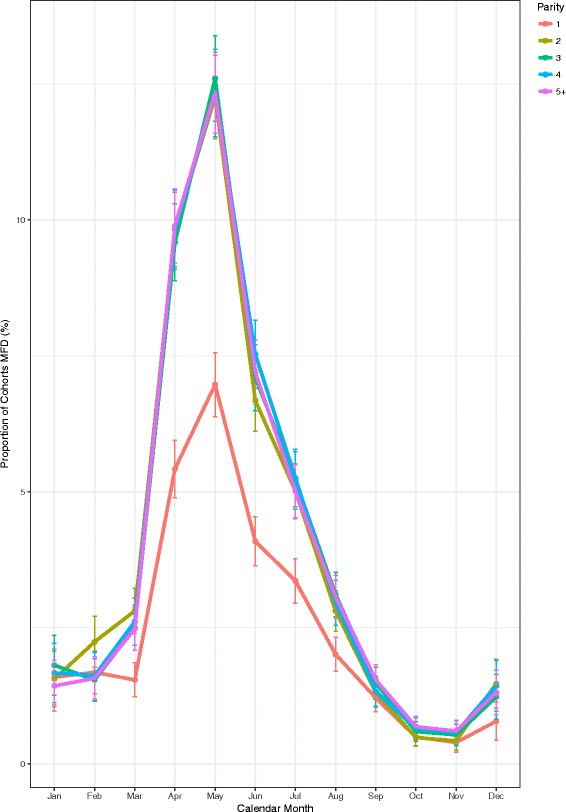
Monthly prevalence of MFD across within-herd cohorts of cows by parity group with error bars showing 95% confidence intervals

The prevalence of MFD in cohorts of cows grouped by parity (Fig. 5) shows that parities ≥2 had similar highest prevalences in May, whereas highest prevalence for parity one cohorts was significantly lower (*p* < 0.001). The highest (May) prevalence of MFD was 7.0% (6.4, 7.6) in parity one cohorts compared to 12.34% (11.6, 14.0) in the parity 5+ cohorts.

**Fig. 6:**
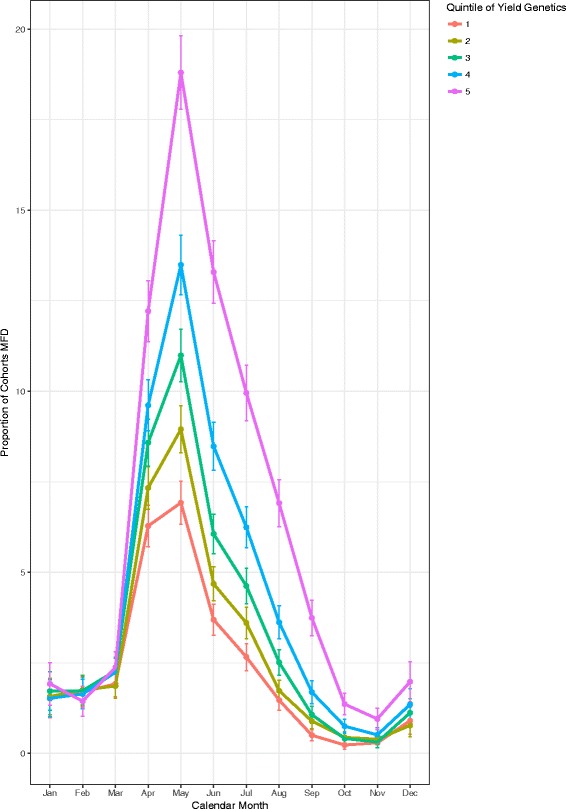
Monthly prevalence of MFD across within-herd cohorts of cows grouped by Quintile of predicted transmitting ability for milk kilograms with error bars showing 95% confidence intervals

**Fig. 7:**
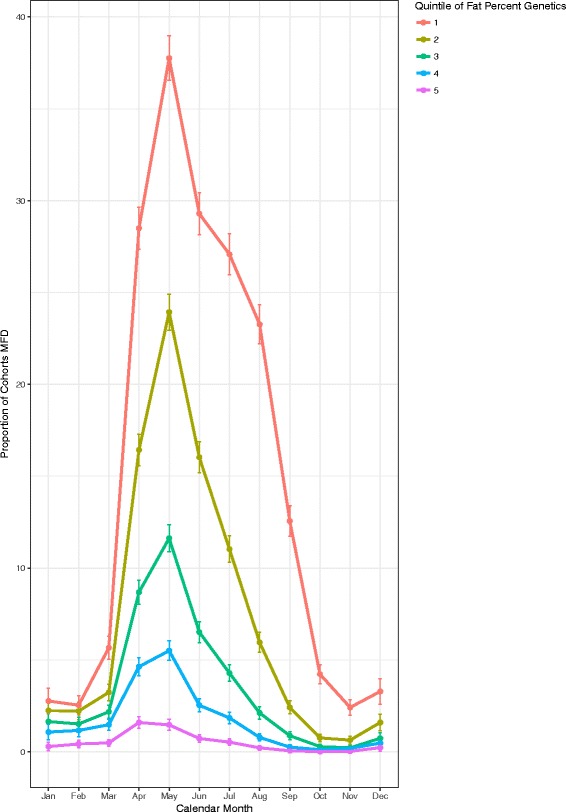
Monthly prevalence of MFD across within-herd cohorts of cows grouped by Quintile of predicted transmitting ability for milk fat percent with error bars showing 95% confidence intervals

Cohorts of cows were grouped by quintile for PTA for milk kg (−23, 76.6, 164, 268, 1437), and the prevalence of MFD over time calculated (Fig. 6). In May, the prevalence of MFD among the cohorts of cows with the lowest PTA value for milk yield also had the lowest prevalence of MFD. There was an 11.9% increase in prevalence from the lowest to the highest PTA quintiles (p < 0.001). Conversely, when the PTA value for milk fat percentage was assessed by quintile (−0.09, −0.02, 0.03, 0.1, 0.84) the reverse of the pattern seen with the yield PTA and MFD was observed (Fig. 7). Cohorts of cows in the lowest quintile for PTA for milk fat percentage had the greatest prevalence of 37.5% (36.3, 38.7), whereas the cohort of cows with the highest quintile for PTA of milk fat percent had a highest prevalence of 1.46% (1.2, 1.8) (*p* < 0.001). The confidence intervals for the highest prevalence of MFD did not overlap for any of the quintile groups.

**Fig. 8:**
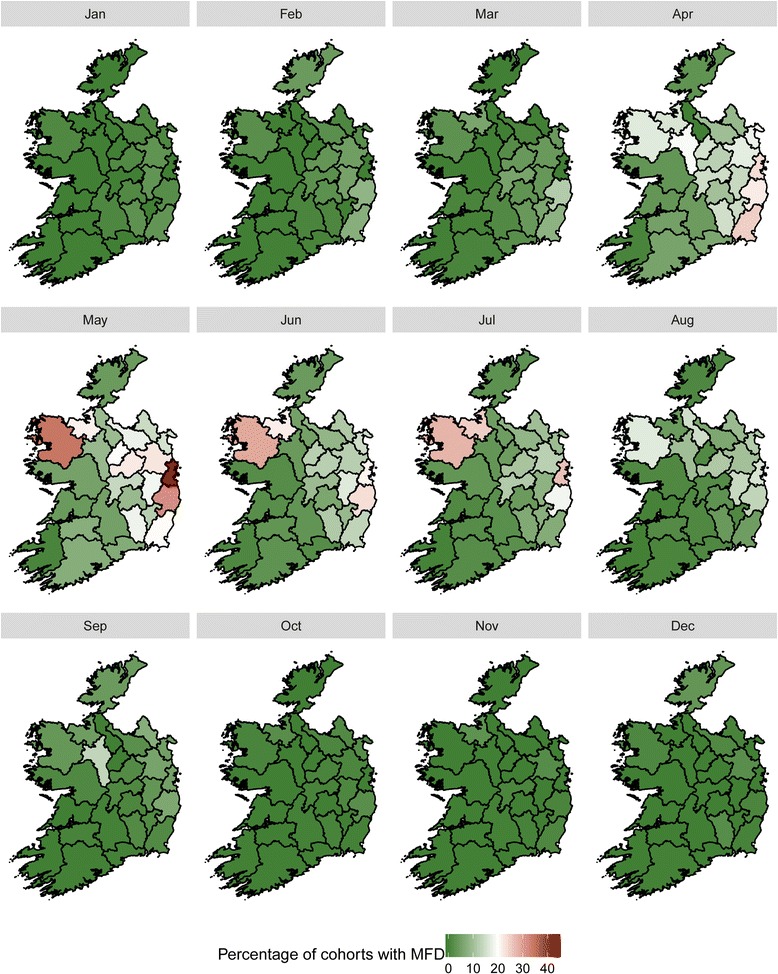
Monthly prevalence of MFD across within-herd cohorts of cows grouped by Spring Calving Season by county

**Fig. 9:**
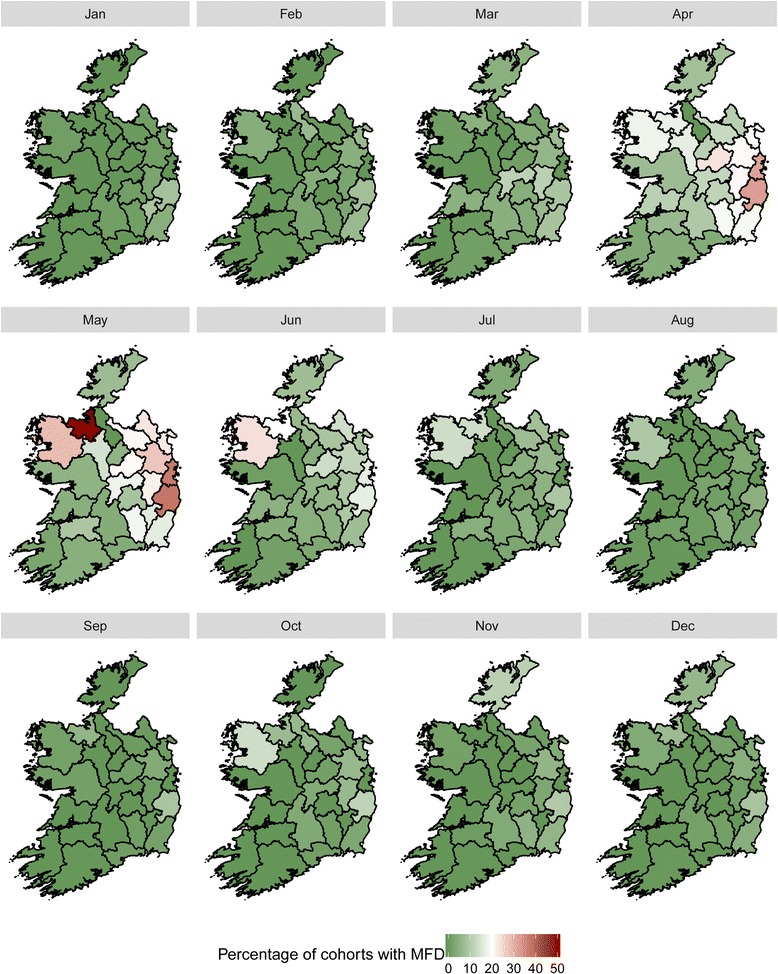
Monthly prevalence of MFD across within-herd cohorts of cows grouped by Autumn Calving Season by county

A spatial trend in MFD was observed. Cohorts of cows within herds were categorised by calving season and prevalence of MFD and plotted graphically across the country in Fig. 8 (spring calved cohorts) and Fig. 9 (autumn calved cohorts). Dublin, Wicklow and Mayo had the highest prevalence of MFD for spring calved cohorts of cows. In the autumn calved cohorts, Dublin, Wicklow, Mayo and Sligo had the highest prevalence of MFD. For both Figs. 8 and 9, the highest prevalence of MFD for both cohorts occurred in May.

## Discussion

This study describes the temporal, spatial and basic animal-level associations with MFD in Irish dairy herds. It has been reported that there is considerable variability in milk fat production including seasonal differences, herd to herd differences and within herd differences [11]. The analyses performed in this study show that overall, the prevalence of MFD has decreased from 2004 to 2014 (Figs. 1 and 2), although there are year to year variations in the prevalence of MFD over time. One potential explanation for this decrease in prevalence over time is that the focus of farmers on milk solid production has greatly increased with milk payment schemes based on milk components now widely adopted by many milk processors in Ireland. The introduction of the EBI system in 2001 is potentially responsible for at least some of this improvement through genetic selection for milk solids [1, 22]. External factors such as changes in grass growth patterns, grass quality or weather patterns may potentially have an environmental impact on the prevalence of MFD.

The increased frequency of MFD in May is consistent throughout the analyses, and had 27 times greater odds of occurring in May compared to the month with the lowest prevalence across the 11 years of data. This finding was consistent, regardless of calving season. This finding may be explained by the observation that this time of year corresponds to peak grass growth and use of grass as a major component of the diet on the majority of Irish farms. High quality perennial ryegrass has been widely implicated as having a causal role in the occurrence of MFD [15]. The seasonal nature of production in Ireland also implies that some spring calving cohorts would be close to peak yield in early lactation in May and consistent with other literature the lowest percentages of fat often occur at this time [10].

Stage of lactation has a direct effect on milk fat production [10]. In a previous study, lowest milk fat percentage was found to occur at day 70 in milk [23]. In the present study, the highest prevalence of MFD in spring calved cows occurred at 61–90 DIM, coinciding with highest prevalence in the month of May. The highest prevalence of MFD in autumn calved cows occurred between 181–210 DIM, which also coincided with the month of May (Figs. 3 and 4). The autumn and the two other calving season cohorts have a similar temporal pattern in prevalence of MFD in Fig. 3. All calving season cohorts have the greatest prevalence of MFD in the month of May, suggesting that time of year is more important for MFD than stage of lactation. The results of Figs. 3 and 4 suggest that prevalence of MFD is due to an environmental influence or an association with the month of May rather than calving season or stage of lactation.

Primiparous cohorts of cows had lower levels of MFD compared to later parities (Fig. 5), the reason for this finding is not clear. The finding in the present study could potentially be explained by lower yields, flatter lactation curve and potentially higher genetic merit in this cohort [24]. In addition, feed intake capacity is lower for parity one cows [25] resulting in lower intake of grass and therefore a decreased intake of PUFA.

Investigation of the association between breeding values and MFD showed that cohorts with the highest PTA value for milk yield had the highest prevalence of MFD, whereas the reverse was observed in cohorts with the highest PTA for fat production. It is not unusual that the higher yielding cows have a higher prevalence of MFD perhaps due a higher potential for intake of PUFA’s. The cows with low values for milk fat percentage had a higher prevalence of MFD. It is interesting that both PTA for yield and milk fat percentage cohorts exhibit the same temporal pattern of MFD. Given that all quintiles are affected along the same temporal pattern it suggests an environmental effect has an influence on occurrence of MFD. It cannot be ruled out that higher PTA for milk fat percent confers some protection from the seasonal effects that precipitate MFD. Further investigation would be required to determine the effect of genetic, environmental factors and interactions between environment and genetics that might influence this outcome.

The spatial patterns in MFD in Ireland are similar among spring and autumn calved cohorts. For both maps, counties Dublin, Wicklow and Mayo feature among the group of counties that had the highest prevalence of MFD. In Ireland, the length of the grazing season can vary between regions and considerable differences in grazing management and therefore in the grass proportion of the diet have been identified [26]. This finding may also be explained through differences in cattle genetics between these areas.

Approximately one third of Irish dairy farmers regularly milk record [20], although due to the seasonal nature of production, the number recording per month varies. The dataset and findings from this study are therefore representative of this subpopulation of herds. Milk recording herds are potentially more likely to represent the most progressive farms within the national herd. It could therefore be argued that the extent of MFD nationally could be even larger. Alternatively, the converse could also be argued; yields could potentially be lower in non-milk recording herds, and associated with a decreased prevalence of MFD. A further limitation of this study is that no data were available on which milk recording method was used. Farmers have the option of ‘do it yourself; DIY’ milk recording or an independent milk recording carried out by a recording technician. However, the method used is unlikely to considerably affect the outcomes of this study.

Finally, although the vast majority of Irish dairy herds are assumed to follow a grazing system, the lack of detailed dietary information and grazing data limited the extent to which changes in grass availability and or quality could be investigated having an effect on MFD.

## Conclusion

This study identified a significant prevalence of MFD in Irish milk recorded herds, with 9.1% of herds affected in May of the most recent year (2014). MFD has reduced over the 11 years of milk records. Despite basic investigation of associated factors, time (month) appeared to have the greatest effect on the occurrence of MFD, likely reflecting the high dietary intake of fresh, high quality grass at this time. Further analyses are required to identify more specifically how the factors investigated are contributing to this problem.

## Additional files

Additional file 1: Table S1. The herd mean, median and mode of test day milk production records between 2004 and 2014. (DOCX 13 kb)

Additional file 2: Table S2. The number of herds milk recording per month between 2004-2014. (DOCX 16 kb)

Additional file 3: Table S3. The number of herds milk recording for each county by month between 2004 and 2014. (DOCX 19 kb)
