# Erratum to: The prevalence, temporal and spatial trends in bulk tank equivalent milk fat depression in Irish milk recorded herds

**DOI:** 10.1186/s13620-017-0098-5

**Published:** 2017-06-13

**Authors:** Catherine I. Carty, Alan G. Fahey, Morgan R. Sheehy, Steve Taylor, Ian J. Lean, Conor G. McAloon, Luke O’Grady, Finbar J. Mulligan

**Affiliations:** 1 School of Veterinary Medicine, University College Dublin, Dublin, Ireland; 2 School of Agricultural Food Science and Nutrition, University College Dublin, Dublin, Ireland; 3 Devenish Nutrition, Lagan House, 19 Clarendon Road, Belfast, Northern Ireland; 4 Scibus Consultancy, 2 Broughton Street, Camden, NSW Australia

## Erratum

Following the publication of this article [1], it was brought to our attention that the PDF version of the article unfortunately contained the following error:

Figure 6 had accidentally been replaced with Figure 5, which incorrectly appeared duplicated in the PDF.

**Fig. 6:**
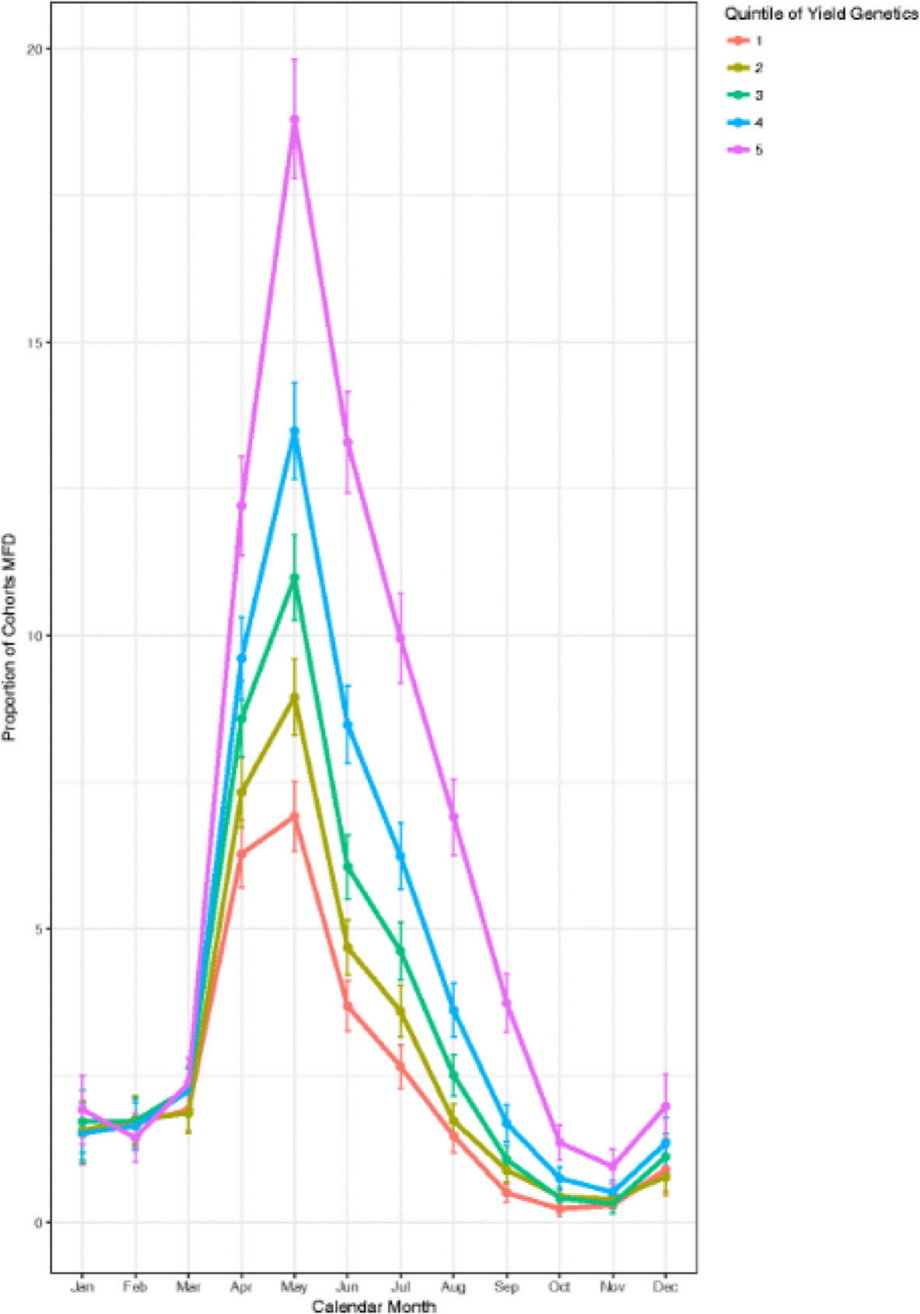
Monthly prevalence of MFD across within-herd cohorts of cows grouped by Quintile of predicted transmitting ability for milk kilograms with error bars showing 95% confidence intervals

The correct Figure 6 is presented below and has been updated in the PDF version of the original article.
