# Supplementary material for: The prevalence, temporal and spatial trends in bulk tank equivalent milk fat depression in Irish milk recorded herds
Source: Ir Vet J. 2017 May 18;70:14. doi: 10.1186/s13620-017-0092-y (PMC5437576; doi:10.1186/s13620-017-0092-y)
Supplement: Supplementary file 1 — The herd mean, median and mode of test day milk production records between 2004 and 2014. (DOCX 13 kb) [file 13620_2017_92_MOESM1_ESM.docx]

**Table 1**

|  | **2004** | **2005** | **2006** | **2007** | **2008** | **2009** | **2010** | **2011** | **2012** | **2013** | **2014** |
| --- | --- | --- | --- | --- | --- | --- | --- | --- | --- | --- | --- |
| **Mean No. Test day records** | 8 | 8 | 7 | 7 | 6 | 6 | 6 | 6 | 6 | 6 | 6 |
| **Median No. Test day records** | 7 | 7 | 7 | 6 | 6 | 6 | 6 | 5 | 5 | 5 | 5 |
| **Mode of Test day records** | 7 | 6 | 4 | 4 | 4 | 4 | 4 | 4 | 4 | 4 | 4 |

The herd mean, median and mode of test day milk production records between 2004 and 2014
