# Supplementary material for: The prevalence, temporal and spatial trends in bulk tank equivalent milk fat depression in Irish milk recorded herds
Source: Ir Vet J. 2017 May 18;70:14. doi: 10.1186/s13620-017-0092-y (PMC5437576; doi:10.1186/s13620-017-0092-y)
Supplement: Supplementary file 2 — The number of herds milk recording per month between 2004-2014. (DOCX 16 kb) [file 13620_2017_92_MOESM2_ESM.docx]

**Table 2**

|  | **Jan** | **Feb** | **Mar** | **Apr** | **May** | **Jun** | **Jul** | **Aug** | **Sep** | **Oct** | **Nov** | **Dec** | **TOTAL** |
| --- | --- | --- | --- | --- | --- | --- | --- | --- | --- | --- | --- | --- | --- |
| **2004** | 1654 | 2813 | 4059 | 4326 | 4383 | 4447 | 4565 | 4651 | 4561 | 4463 | 3693 | 2203 | **45818** |
| **2005** | 2136 | 2924 | 4022 | 4133 | 4366 | 4100 | 4222 | 4254 | 4053 | 4234 | 3454 | 1916 | **43814** |
| **2006** | 1963 | 2520 | 3633 | 4005 | 4106 | 3937 | 3991 | 4054 | 3807 | 4148 | 3452 | 1778 | **41394** |
| **2007** | 1714 | 2228 | 3421 | 4006 | 3819 | 3837 | 3814 | 3880 | 3511 | 4143 | 2901 | 1592 | **38866** |
| **2008** | 1582 | 2193 | 3029 | 3840 | 3700 | 3700 | 3664 | 3743 | 3411 | 3957 | 2501 | 1492 | **36812** |
| **2009** | 1374 | 1850 | 2894 | 3372 | 3098 | 3338 | 3288 | 3312 | 3110 | 3457 | 2247 | 1211 | **32551** |
| **2010** | 1175 | 1806 | 2875 | 3529 | 3308 | 3314 | 3355 | 3403 | 3329 | 3453 | 2515 | 1231 | **33293** |
| **2011** | 1231 | 1741 | 2955 | 3410 | 3241 | 3387 | 3225 | 3550 | 3243 | 3376 | 2171 | 1146 | **32676** |
| **2012** | 1115 | 1838 | 3004 | 3363 | 3489 | 3252 | 3371 | 3442 | 3165 | 3711 | 2220 | 1082 | **33052** |
| **2013** | 1014 | 1828 | 2596 | 3377 | 3555 | 3225 | 3505 | 3521 | 3128 | 3829 | 2262 | 1110 | **32950** |
| **2014** | 1001 | 1574 | 2706 | 3559 | 3378 | 3290 | 3468 | 3498 | 3173 | 3840 | 1987 | 919 | **32393** |

The number of herds milk recording per month between 2004-2014
