# Supplementary material for: The prevalence, temporal and spatial trends in bulk tank equivalent milk fat depression in Irish milk recorded herds
Source: Ir Vet J. 2017 May 18;70:14. doi: 10.1186/s13620-017-0092-y (PMC5437576; doi:10.1186/s13620-017-0092-y)
Supplement: Supplementary file 3 — The number of herds milk recording for each county by month between 2004 and 2014. (DOCX 19 kb) [file 13620_2017_92_MOESM3_ESM.docx]

|  | **Jan** | **Feb** | **Mar** | **Apr** | **May** | **Jun** | **Jul** | **Aug** | **Sep** | **Oct** | **Nov** | **Dec** |  | **Total** |
| --- | --- | --- | --- | --- | --- | --- | --- | --- | --- | --- | --- | --- | --- | --- |
| **Carlow** | 35 | 38 | 51 | 70 | 58 | 67 | 67 | 69 | 65 | 69 | 57 | 38 |  | 684 |
| **Cavan** | 193 | 213 | 245 | 264 | 272 | 271 | 274 | 271 | 278 | 274 | 267 | 210 |  | 3032 |
| **Clare** | 15 | 28 | 133 | 199 | 215 | 216 | 208 | 216 | 211 | 222 | 112 | 25 |  | 1800 |
| **Cork** | 1004 | 1846 | 2510 | 2749 | 2739 | 2739 | 2741 | 2741 | 2707 | 2748 | 2227 | 1263 |  | 28014 |
| **Donegal** | 26 | 27 | 33 | 64 | 73 | 69 | 72 | 77 | 69 | 74 | 70 | 30 |  | 684 |
| **Dublin** | 19 | 19 | 19 | 19 | 19 | 19 | 19 | 19 | 19 | 19 | 19 | 19 |  | 228 |
| **Galway** | 73 | 81 | 122 | 149 | 150 | 144 | 147 | 147 | 144 | 156 | 114 | 80 |  | 1507 |
| **Kerry** | 162 | 407 | 645 | 721 | 734 | 717 | 734 | 733 | 721 | 728 | 575 | 244 |  | 7121 |
| **Kildare** | 75 | 81 | 86 | 87 | 90 | 88 | 89 | 87 | 87 | 88 | 87 | 78 |  | 1023 |
| **Kilkenny** | 81 | 178 | 254 | 312 | 308 | 318 | 300 | 317 | 301 | 322 | 211 | 100 |  | 3002 |
| **Laois** | 75 | 112 | 158 | 195 | 186 | 195 | 188 | 202 | 190 | 203 | 156 | 92 |  | 1952 |
| **Leitrim** | 17 | 17 | 20 | 18 | 21 | 19 | 21 | 20 | 20 | 20 | 18 | 20 |  | 231 |
| **Limerick** | 138 | 331 | 574 | 664 | 660 | 660 | 667 | 670 | 668 | 679 | 477 | 187 |  | 6375 |
| **Longford** | 32 | 35 | 48 | 65 | 59 | 64 | 66 | 62 | 61 | 62 | 57 | 38 |  | 649 |
| **Louth** | 96 | 98 | 106 | 107 | 107 | 107 | 108 | 104 | 105 | 103 | 101 | 87 |  | 1229 |
| **Mayo** | 76 | 89 | 106 | 116 | 112 | 118 | 118 | 119 | 106 | 124 | 105 | 85 |  | 1274 |
| **Meath** | 279 | 277 | 309 | 306 | 313 | 306 | 309 | 304 | 303 | 294 | 300 | 256 |  | 3556 |
| **Monaghan** | 161 | 161 | 181 | 192 | 189 | 194 | 190 | 197 | 194 | 190 | 187 | 164 |  | 2200 |
| **Offaly** | 51 | 80 | 126 | 159 | 150 | 158 | 152 | 162 | 149 | 163 | 107 | 62 |  | 1519 |
| **Roscommon** | 9 | 13 | 17 | 26 | 23 | 25 | 29 | 21 | 23 | 26 | 17 | 10 |  | 239 |
| **Sligo** | 25 | 28 | 36 | 48 | 43 | 47 | 45 | 48 | 43 | 47 | 36 | 24 |  | 470 |
| **Tipperary** | 98 | 234 | 522 | 695 | 706 | 711 | 696 | 722 | 666 | 742 | 419 | 133 |  | 6344 |
| **Waterford** | 97 | 189 | 304 | 358 | 355 | 355 | 361 | 361 | 346 | 349 | 244 | 106 |  | 3425 |
| **Westmeath** | 78 | 89 | 102 | 120 | 114 | 120 | 111 | 121 | 113 | 124 | 103 | 85 |  | 1280 |
| **Wexford** | 213 | 286 | 357 | 382 | 381 | 382 | 390 | 390 | 373 | 382 | 316 | 225 |  | 4077 |
| **Wicklow** | 109 | 120 | 122 | 129 | 126 | 131 | 132 | 131 | 132 | 130 | 126 | 112 |  | 1500 |

**Table 3**

The number of herds milk recording for each county by month between 2004 and 2014
